# Complete chloroplast genome sequence of *Acer sutchuenense* subsp. *tienchuanenge* (Sapindaceae)

**DOI:** 10.1080/23802359.2020.1791002

**Published:** 2020-07-20

**Authors:** Wei Zhang, Xinhe Xia, Difei Chen, Jiayong Li, Wei He, Wenbao Ma, Bixian Wu, Yongqi Zheng, Chuanhong Zhang

**Affiliations:** a State Key Laboratory of Tree Genetics and Breeding, Laboratory of Forest Silviculture and Tree Cultivation, Research Institute of Forestry, Chinese Academy of Forestry, Beijing, P. R. China; b Sichuan Academy of Forestry, Chengdu, P. R. China; c Faculty of Forestry, Sichuan Agricultural University, Wenjiang, P. R. China

**Keywords:** Chloroplast genome, *Acer sutchuenense* subsp. *tienchuanenge*, phylogenetic analysis

## Abstract

*Acer sutchuenense* subsp. *tienchuanenge* (Sapindaceae: *Acer*) is an endangered deciduous arbor species and endemic to China. Being obtained by using genome Illumina pair-end sequencing data, the complete chloroplast genome of *A. sutchuenense* subsp. *tienchuanenge* had a typical quadripartite structure, with 156,063 bp long, including a large single-copy (LSC) region of 85,772 bp, a small single-copy (SSC) region of 18,117 bp, and a pair of inverted repeats (IRs) (each 26,087 bp in length). A total of 136 genes were annotated, of which 113 are unique genes, including 30 tRNAs, 4 rRNAs, and 79 protein-coding genes. The overall GC content was 37.9%. The phylogenetic analysis suggested that *A. sutchuenense* subsp. *tienchuanenge* was the most closely related to *A. griseum* and *A. triflorum.* The complete chloroplast genome of *A. sutchuenense* subsp. *tienchuanenge* is valuable for assessment and conservation of genetic resources and further for phylogenetic study of *Acer* L.

*Acer sutchuenense* has two subspecies, which are *A. sutchuenense* subsp. *sutchuenense* and *A. sutchuenense* subsp. *tienchuanenge*. *Acer sutchuenense* subsp. *tienchuanenge* is a deciduous arbor species, mainly distributed in west and southwest of Sichuan province, while *A. sutchuenense* subsp. *sutchuenense* is distributed in Chongqing municipality, west of Hubei province and southwest of Hunan province (Wu et al. 2008). Except for the difference of geographical distribution, the trunk and leaves of *A. sutchuenense* subsp. *tienchuanenge* are larger than those of *A. sutchuenense* subsp. *sutchuenense*. According to the China Species Red List, *A. sutchuenense* was Critically Endangered (EN) (Wang and Xie 2004; Gibbs and Chen 2009). Till now only a few cases of study on investigation of wild resources and taxonomic status of *A. sutchuenense* (Zhang et al. 2011; Jiang et al. 2017; Wei 2019). Here, the complete chloroplast genome sequence of *A. sutchuenense* subsp. *tienchuanenge* was reported to provide a genomic resource and to clarify phylogenetic relationship of this plant with the other species in the Sapindaceae family.

Fresh leaves of *A. sutchuenense* subsp. *tienchuanenge* were collected from a wild individual tree from the Mount Emei, Sichuan province, China (N29°32′55′′, E103°21′28′′). The voucher specimen was deposited in Laboratory of Forest Silviculture and Tree Cultivation, Research Institute of Forestry, Chinese Academy of Forestry in Beijing, China (Voucher specimen: ACSUTCH-SCEM2019-01). The plant genomic DNA extraction kit (DP350) (Tiangen biotech Inc., Beijing, China) was used to extract the total genomic DNA, which was sequenced using the Illumina Hiseq platform (Huitong biotechnology Inc., Shenzhen, China). SPAdes version 3.9.0 and DOGMA were used for de novo assembly and annotation of the chloroplast genome (Wyman et al. 2004; Bankevich et al. 2012). The chloroplast genome map was drawn by using OGDRAW (Lohse et al. 2013).

The whole chloroplast genome of *A. sutchuenense* subsp. *tienchuanenge* had a typical quadripartite structure, with 156,063 bp long, including a large single-copy (LSC) region of 85,772 bp, a small single-copy (SSC) region of 18,117 bp, and a pair of inverted repeats (IRs) (each 26,087 bp in length). The total GC content is 37.9%. A total of 136 genes were annotated, of which 113 are unique genes, including 30 tRNAs, 4 rRNAs, and 79 protein-coding genes. The complete cp genome characteristics of *A. sutchuenense* subsp. *tienchuanenge* are similar to that of *A. griseum* (Wang et al. 2017).

Twenty one complete chloroplast genomes were downloaded from NCBI Organelle Genome Database in order to reveal the phylogenetic position of *A. sutchuenense* subsp. *tienchuanenge*. Based on the GTR model, maximum-likelihood analysis and reconstruction of the phylogenetic relationship of 22 species were performed by MEGA version 7.0.14 (Kumar et al. 2016) (Figure 1). All sequences were aligned using MAFFT (Nakamura et al. 2018). The results showed that *A. griseum* and *A. triflorum* were most closely related, followed by *A. sutchuenense* subsp. *tienchuanenge*, which was consistent with the morphological taxonomy, but inconsistent with the results of Wei (2019), which indicated that *A. sutchuenense* and *A. triflorum* were the most closely related. In addition, 14 *Acer* species were clustered into a monophyly by 100% bootstrap value and have the closest relationship with *Dipteronia*, consistent with the result of Xia et al. (2020). In short, the obtained chloroplast genome information of *A. sutchuenense* subsp. *tienchuanenge* was useful for the phylogenetic study of *Acer* L.

**Figure 1.**
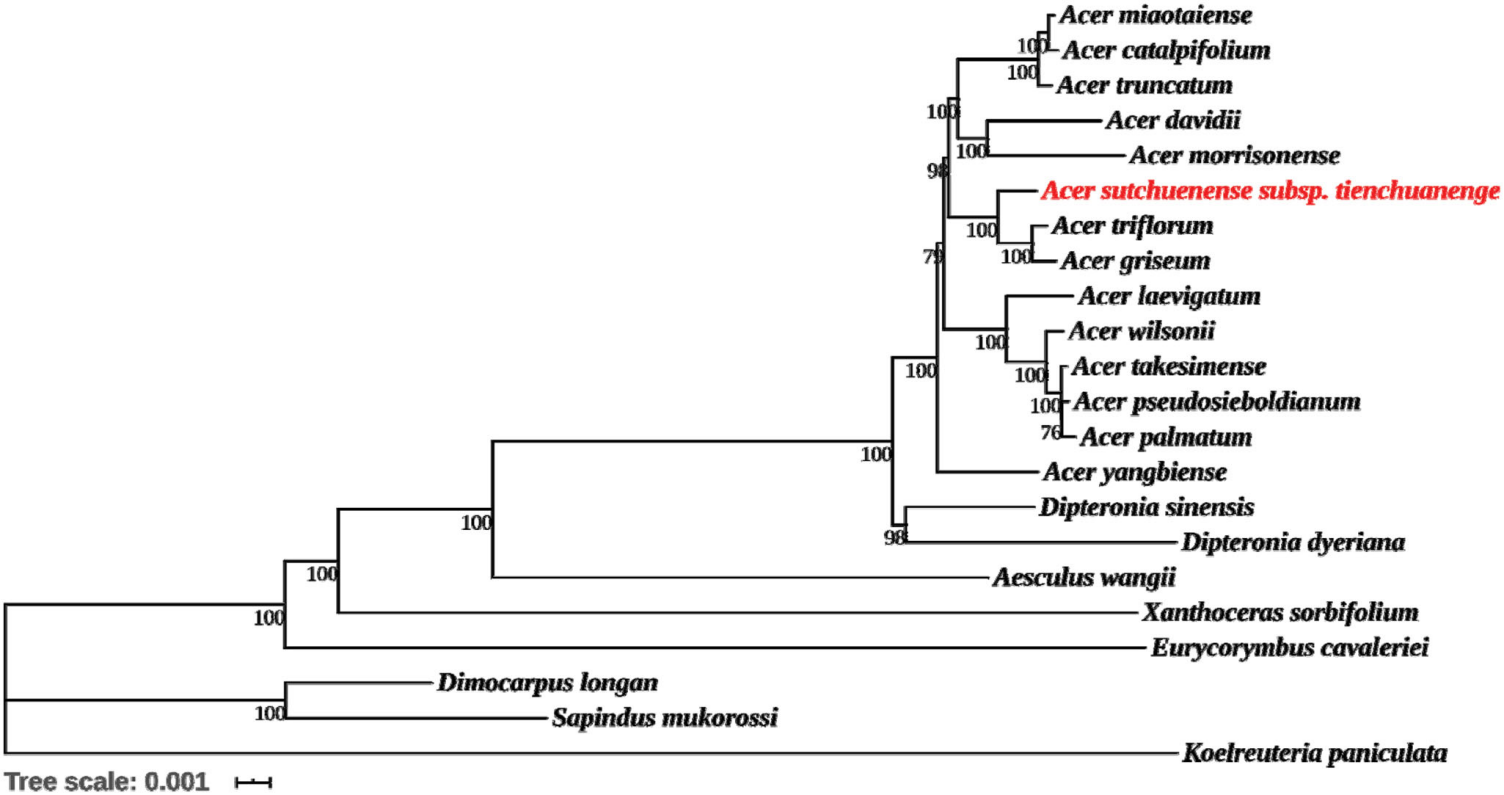
Phylogenetic tree reconstruction of 22 species using maximum likelihood (ML) based on the complete chloroplast genome sequences. There are the bootstrap support values from 1000 replicates given at each node. Their accession numbers are as follows: *Acer catalpifolium*: NC_041080; *Acer davidii*: NC_030331; *Acer griseum*: NC_034346; *Acer pseudosieboldianum*: NC_046487; *Acer laevigatum*: NC_042443; *Acer miaotaiense*: NC_030343; *Acer morrisonense*: NC_029371; *Acer palmatum*: NC_034932; *Acer takesimense*: NC_046488; *Acer triflorum*: NC_047296; *Acer truncatum*: NC_037211; *Acer wilsonii*: NC_040988; *Acer yangbiense*: MK479229; *Aesculus wangii*: NC_035955; *Dimocarpus longan*: NC_037447; *Dipteronia dyeriana*: NC_031899; *Dipteronia sinensis*: NC_029338; *Eurycorymbus cavaleriei*: NC_037443; *Koelreuteria paniculata*: NC_037176; *Sapindus mukorossi*: NC_025554; *Xanthoceras sorbifolium*: NC_037448.

## Data Availability

The data that support the findings of this study are openly available in GenBank of NCBI at https://www.ncbi.nlm.nih.gov/, reference number MT216764.
